# Gene Disruption of *Plasmodium falciparum p52* Results in Attenuation of Malaria Liver Stage Development in Cultured Primary Human Hepatocytes

**DOI:** 10.1371/journal.pone.0003549

**Published:** 2008-10-28

**Authors:** Ben C. L. van Schaijk, Chris J. Janse, Geert-Jan van Gemert, Melissa R. van Dijk, Audrey Gego, Jean-Francois Franetich, Marga van de Vegte-Bolmer, Samir Yalaoui, Olivier Silvie, Stephen L. Hoffman, Andrew P. Waters, Dominique Mazier, Robert W. Sauerwein, Shahid M. Khan

**Affiliations:** 1 Department of Medical Microbiology, Radboud University Nijmegen Medical Centre, Nijmegen, The Netherlands; 2 Department of Parasitology, Leiden University Medical Centre, Leiden, The Netherlands; 3 INSERM, U511, Paris, France; 4 Université Pierre et Marie Curie-Paris6, UMR S511 Paris, France; 5 Sanaria Inc., Rockville, Maryland, United States of America; 6 AP-HP, Groupe hospitalier Pitié-Salpêtrière, Service Parasitologie-Mycologie, Paris, France; Walter and Eliza Hall Institute of Medical Research, Australia

## Abstract

Difficulties with inducing sterile and long lasting protective immunity against malaria with subunit vaccines has renewed interest in vaccinations with attenuated *Plasmodium* parasites. Immunizations with sporozoites that are attenuated by radiation (RAS) can induce strong protective immunity both in humans and rodent models of malaria. Recently, in rodent parasites it has been shown that through the deletion of a single gene, sporozoites can also become attenuated in liver stage development and, importantly, immunization with these sporozoites results in immune responses identical to RAS. The promise of vaccination using these genetically attenuated sporozoites (GAS) depends on translating the results in rodent malaria models to human malaria. In this study, we perform the first essential step in this transition by disrupting, *p52*, in *P. falciparum* an ortholog of the rodent parasite gene, *p36p*, which we had previously shown can confer long lasting protective immunity in mice. These *P. falciparum* P52 deficient sporozoites demonstrate gliding motility, cell traversal and an invasion rate into primary human hepatocytes *in vitro* that is comparable to wild type sporozoites. However, inside the host hepatocyte development is arrested very soon after invasion. This study reveals, for the first time, that disrupting the equivalent gene in both *P. falciparum* and rodent malaria *Plasmodium* species generates parasites that become similarly arrested during liver stage development and these results pave the way for further development of GAS for human use.

## Introduction

*Plasmodium falciparum* is the human parasite responsible for the vast majority of deaths associated with malaria, estimated to be between 1–2 million per year [1]. Drug resistant parasite strains, insecticide resistant mosquitoes and the lack of adequate global control measures have meant that malaria continues to be a major international health issue [2]. Despite years of effort on testing a variety of sub-unit vaccines designed to a variety of antigens expressed at various stages of the parasite life-cycle, success has been limited [3]–[5]. The complexity of both the parasites life-cycle and host immune responses to infection have contributed to the slow progress in the development of a vaccine that can induce efficient and long lasting protective immune responses [6]. Recently, there has been a renewed interest in the attenuated whole-organism vaccine strategy [7]. Initially, this approach has used radiation-attenuated sporozoites (RAS) to obtain sterile immunity experimentally in both mice and humans [8], [9]. Specifically, full protective immunity against *Plasmodium* infection was achieved by immunisation only with live attenuated sporozoites (the infectious form of the parasite injected by the mosquito) that invade and then abort development inside hepatocytes in the liver of both rodent models of malaria and in humans [10].

Recently, it has been shown that a comparable attenuation of liver stage development can be achieved either by the targeted deletion of specific genes that are essential for liver stage development generating genetically attenuated sporozoites (GAS; [11]–[15]) or by chemical attenuation of sporozoites (CAS) [16]. In rodent models, GAS and CAS resemble both RAS and wild-type parasites in terms of invasion of host hepatocytes but, like RAS, they abort and/or arrest development inside the hepatocyte. Importantly, immunisation with both GAS and CAS also induce sterile immunity that is comparable to RAS.

Attenuation by genetic modification may have several advantages compared to CAS and RAS in that it generates parasites with a defined attenuation and results in homogeneous population of parasites. This, therefore, removes any issues with the delivery of correct doses of either irradiation or drugs in order to obtain precisely attenuated parasites that both invade hepatocytes and also become developmentally arrested [17].

Recently, GAS have been produced in the rodent malaria parasites, *P. berghei and P. yoelii*, by single gene deletion of a number of genes (*uis3*, *uis4*, *sap1* and *p36p*) as well as the simultaneous deletion of two genes (*p52+p36* in *P. yoelii*; *uis3+uis4* in *P. berghei* [11]–[15], [18], [19]). Immunisation with sporozoites of all these resulting parasite lines induce, to varying degrees, protection against re-infection with wild type parasites. Studies on these parasites show that they are sufficient to confer protection in some cases with doses as low as 1000–10000 sporozoites [18], [20].

In our laboratory we have generated attenuated *P. berghei* sporozoites by deleting the gene encoding *p36p*. This protein is a member of a small family of proteins that is conserved in *Plasmodium* [21], which includes some important antigens which are putative-candidates for transmission blocking vaccines (i.e. P48/45, P*230*; [22]–[26]). Sporozoites, deficient in expressing P36p resulted in aborted development in hepatocytes, prior to parasite replication. Immunisation with Δ*pb36p* sporozoites induces long lasting and protective immune responses against challenge with wild-type sporozoites in rodents [15] and confers a degree of cross-species protection against other rodent parasites [20]. It has also been shown in *P. yoelii* that the disruption of the ortholog of *p36p* and its paralogous gene, *p36*, results in generation of attenuated sporozoites that can confer protective immunity [18].

In order to translate the promising observations in rodent models of malaria to humans, that GAS have the capacity to induce protective immune responses comparable to RAS, it is first necessary to generate *P. falciparum* mutants that are also attenuated during liver stage development.

In this study, we therefore generated *P. falciparum* parasites that were deficient in expressing P52 (PFD0215c), the equivalent of *P. berghei* P36p. The analysis of sporozoite invasion of hepatocytes *in vitro* as well as development within primary human hepatocytes with *P. falciparum* Δ*p52* mutants demonstrates a pattern of attenuation essentially identical to *P. berghei* mutants unable to express P36P. Specifically, development aborts shortly after hepatocyte invasion. These findings open up the exciting possibility that, as with the *P. berghei* Δ*p36p* sporozoites, *P. falciparum* mutants lacking this gene may also confer protective immunity in humans against wild-type sporozoite infection.

## Results

### The *P. falciparum p52* gene (PFD0215c) is an ortholog of *P. berghei p36p* (PB000891.00.0) and is amenable to gene disruption

In the *P. berghei* genome the two neighbouring genes *p36* (PB000892.00.0) and *p36p* (PB000891.00.0) are a paralogous pair of genes located on chromosome 10 and based on sequence similarity (i.e. 46% amino acid sequence similarity). These genes belong to a larger gene family constituting 10 members i.e. the 6-cys family [21]. The repertoire of genes within this gene family is similarly expanded within all (currently sequenced) genomes of *Plasmodium* with every member of the *P. berghei* gene family having a direct ortholog in *P. falciparum* based both on sequence similarity and syntenic positioning of genes [21]. Previously, it has been described that the expression of *P. berghei 36p* appears to be exclusive to the sporozoite stage [27]–[29], which is supported by the presence of P36p peptides only in the proteome of *P. berghei* sporozoites [30], detection of the protein by Western analysis of proteins of salivary gland sporozoites (SGS) [31] and the presence of transcripts in *P. berghei* SGS [32]. Further, this stage specific expression was also observed for the orthologous protein in the closely related rodent malaria parasite, *P. yoelii*, where both protein and transcripts are present in the SGS stage [33].

The ortholog of *P. berghei p36p* in *P. falciparum* is PFD0215c, referred to as *p52* [29] and (www.PlasmoDB.org), they share 39% amino acid sequence identity (and 58% similarity) as well as the corresponding syntenic conservation (*P. falciparum* chromosome 4 and *P. berghei* chromosome 10; [34]). Examination of the available *P. falciparum* proteomes reveals that peptides corresponding to this protein are only detected in the SGS proteome of Lasonder et al 2008 (i.e. 5 unique peptides) and also transcriptome analyses indicate that expression only occurs in SGS [35].

To investigate if a *P. falciparum* mutant lacking the *p52* gene would also manifest the same attenuated phenotype during development in the liver, as observed with *P. berghei* mutants lacking *p36p*, two independent transfections were performed to disrupt *p52* in *P. falciparum*.

**Figure 1:**
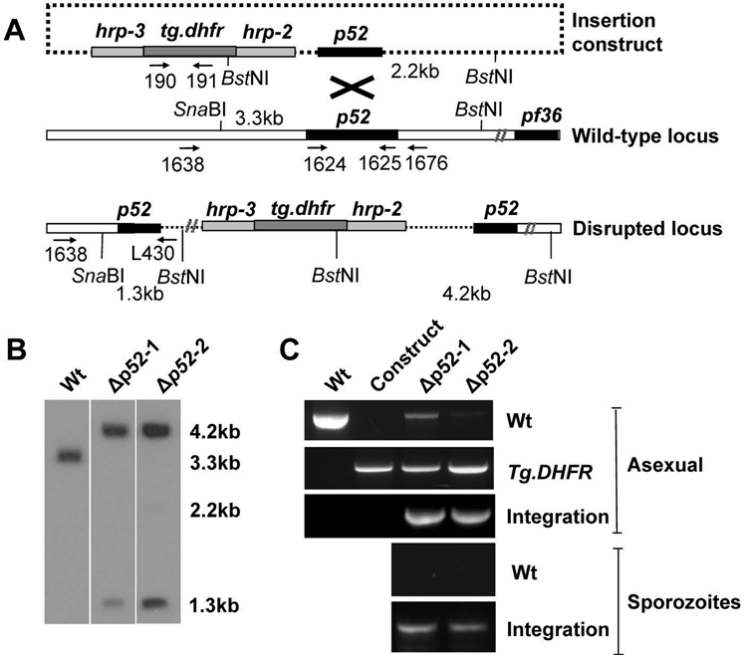
Generation of *P. falciparum* parasites lacking expression of P52. (A) Illustration of the DNA construct (m144) used for the targeted gene disruption of *p52* and the *p52*-genomic locus before and after integration. Shown are the p52 gene and target sequence (amplified using 1624 & 1625), the paralog of p52, p36, and the *T. gondii dhfr/ts* selection cassette. In addition, primer pairs and restriction sites for diagnostic PCR and Southern analysis are shown (see B and C). hrp – histidine rich protein. (B) Southern analysis of *BstN*I/*SnaB*I digested genomic DNA of Wt and *Δp52* demonstrates correct disruption of *p52*. DNA was hybridized with a *p52* specific probe detecting a 3.3 kb fragment in Wt, a 2.2 kb fragment for intact plasmid and the expected fragments of 1.3 kb and a 4.2 kb band (see A) in the two *Δp52* clones (*Δp52-1 and Δp52 -2*). (C) PCR analysis of genomic DNA of Wt and *Δp52* clones and the plasmid DNA (construct) demonstrates correct disruption of *p52*. Genomic DNA from Wt and *Δp52* asexual parasites and sporozoites was used as template for the PCR reactions. The Wt specific PCR was performed using primers 1638 and 1676 amplifying a 2.1 kb fragment. PCR primer pairs 1638 and L430, specific for integration of the DNA construct (see A) amplify a 2.0 kb fragment. Primer pairs 190 and 191 amplifying a 1.8 kb fragment from *T. gondii dhfr/ts* were used as a control.

The construct contained the *Toxoplasma gondii* DHFR selection cassette and a 1020 base pair internal fragment of the *p52* coding sequence that is used as target sequence for integration of the construct into the *P. falciparum p52* locus by single cross-over integration (see Figure 1A for details/schematic representation of the construct and the integration event). Blood stage parasites of the NF54 strain of *P. falciparum* were transfected as previously described [36] and pyrimethamine resistant parasites were selected by standard methods for drug-selection of transformed *P. falciparum* parasites. Cloned lines of the resistant parasite populations were obtained for both experiments (i.e. clone *Δp52-1 and* Δ*p52-2*) by the method of limiting dilution. Correct integration of the construct and disruption of the *p52* locus was demonstrated for one clone of each line by diagnostic PCR and Southern analysis of restricted DNA (Figure 1B&C). Since we have used a construct designed for single cross-over integration, reversion of the disrupted locus to wild type can occur at low frequency in the parasite population as has been reported for *P. berghei* TRAP mutants [37]. It is possible that such reversion events can be detected by sensitive PCR analysis resulting in low amounts of wild type PCR fragments (Figure 1C).

### The Δ*p52* parasites have comparable development to wild-type parasites during blood stage growth, in culture, and in the mosquito

During the cloning procedure of the mutant parasites and subsequent *in vitro* cultivation of the asexual blood stages, the growth and multiplication characteristics of the two mutant clones, Δ*p52-1* and Δ*p52-2*, were comparable to wild type parasites of the parent line NF54 (data not shown).

Gametocyte production of the mutant parasites was analysed in blood stage cultures that were optimised for gametocytogenesis [38]. Gametocyte production of the mutant parasites ranged between 14 and 87 gametocytes/1000 erythrocytes which is comparable to wild type gametocyte production (Table 1) and gametocytes were able to develop in morphologically mature (stage V) parasites with a similar morphology to wild type parasites [39]. Male gametocytes were functionally mature as shown by exflagellation (formation of gametes) *in vitro* (Table 1) and formed the characteristic exflagellation centres after induction of gametogenesis.

**Table 1:** Gametocyte, oocyst and sporozoite development of Δ*p52* parasites.

Parasite	Gametocyte no. Per 1000 RBC (range)	Exfl.^a^	Oocyst production^b^ (IQR)	Infected/dissected mosquitoes	% Infected mosquitoes	Mean no. of sporozoites per mosquito (std)
Wt	27 (12–50)	+	22 (6/39)	36/40	90	55 633 (22.580)
Δ*p52-1*	27 (14–36)	+	13 (4/26)	35/40	88	44 632 (9.953)
Δ*p52-2*	38 (12–87)	+	23 (5/51)	37/40	93	76 746 (30.339)

a Exflagellation (Exfl) of male gametocytes was determined in small samples from the cultures by counting exflagellation centres under the light-microscope in 25 homogeneous fields of rbc at a 40× magnification. A mean of 2–10 per field is scored as +; >10 as ++ and less then 2 as +/−.

b Oocyst production is the median of the oocysts counted at day 7 after mosquito feeding and IQR is the inter quartile range. No significant difference exist between mutant and wild-type parasites (Wilcoxin rank-sum test; p = 0.13 for Δ*p52-1* and p = 0.5 for Δ*p52-2*).

Parasite development in the mosquito was analysed by feeding female *A. stephensi* mosquitoes using standard membrane feeding of cultured gametocytes [38] and subsequent monitoring of oocyst and sporozoite production. Counting of oocysts at day 7 showed that the mutant lines produced infections in 88–93% of the mosquitoes with oocyst numbers ranging from 4–52 per mosquito which is comparable to wild type mosquito infection (Table 1). Also the sporozoite production with a mean number per mosquito of 44.632 and 76.764 for Δ*p52-1 and* Δ*p52-2* respectively, was also similar to wild type (Table 1).

### Sporozoites of Δ*p52* parasites have gliding motility and a traversal capacity comparable to wild-type sporozoites

**Figure 2:**
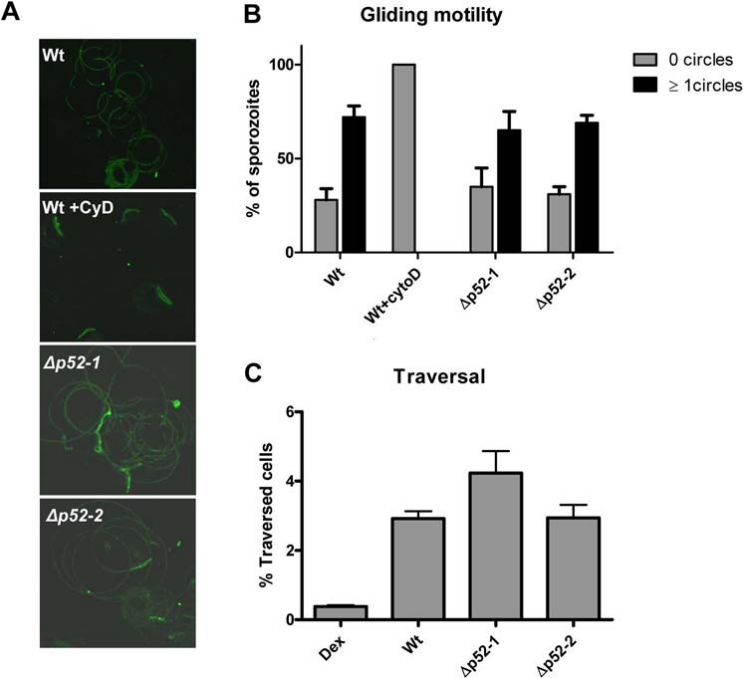
Gliding Motility and Traversal Capacity of Wt and Δ*p52* sporozoites. (A) Representative immunofluorescence staining with anti-PfCSP antibodies of the trails produced by Wt and mutant sporozoites deficient in P52 expression (Δ*p52-1 and* Δ*p52-2*) as well as Wt sporozoites, treated with cytochalasin D, an inhibitor of sporozoite motility. Characteristic circles of gliding motility are present in Wt and mutant lines, and absent in Wt sporozoites that have been treated with cytochalasin D. (B) Gliding motility of *P. falciparum* Wt (cytochalsin D treated and untreated) and mutant sporozoites as assessed by the capacity to produce the characteristic circles (see A). (C) Cell traversal ability of *P. falciparum* Wt and mutant sporozoites as determined by FACS counting of Dextran positive hepG2 cells. Dex: hepatocytes cultured in the presence of Dextran but without the addition of sporozoites.

The ability of mutant sporozoites to move by gliding motility is essential for invasion and was assessed by their ability to ‘glide’ on glass slides [40]. The motility of Δ*p52-1*, Δ*p52-2* and NF54 (Wt) parasites was visualised by counterstaining the trails left by the sporozoites with anti-PfCSP1 antibodies and quantifying the amount of sporozoites out of 100 sporozoites that left trails. This analysis showed that sporozoites of both mutant-lines are able to glide and produce the repeating circles characteristic of correct gliding (Figure 2A) and, moreover, gliding motility is comparable to wild type parasites (Figure 2B).

It has been shown that *Plasmodium* sporozoites migrate to the liver and then traverse/transmigrate through several hepatocytes before they establish an infection in a hepatocyte residing inside a parasitophorous vacuole [41], [42]. To determine if the lack of P52 expression has an effect on sporozoites cell traversal, we analysed hepatocyte traversal *in vitro* using the Dextran incorporation FACS assay as previously described [43]. Only wounded cells incorporate Dextran and by quantifying these cells by FACS, we were able to demonstrate that sporozoites of both mutant lines have a cell traversal rate in cultured hepatoma cells (hepG2) that is comparable to that of wild type parasites. On average Δ*p52-1* migrated through 4.2% of cells, Δ*p52-2* through 2.9% of cells and wild-type through 2.9% hepatocytes as compared to the Dextran only control where only 0.38% cells were Dextran positive (Figure 2C).

### The Δ*p52* parasites are arrested early during hepatocyte development in cultured primary human hepatocyte cells

**Figure 3:**
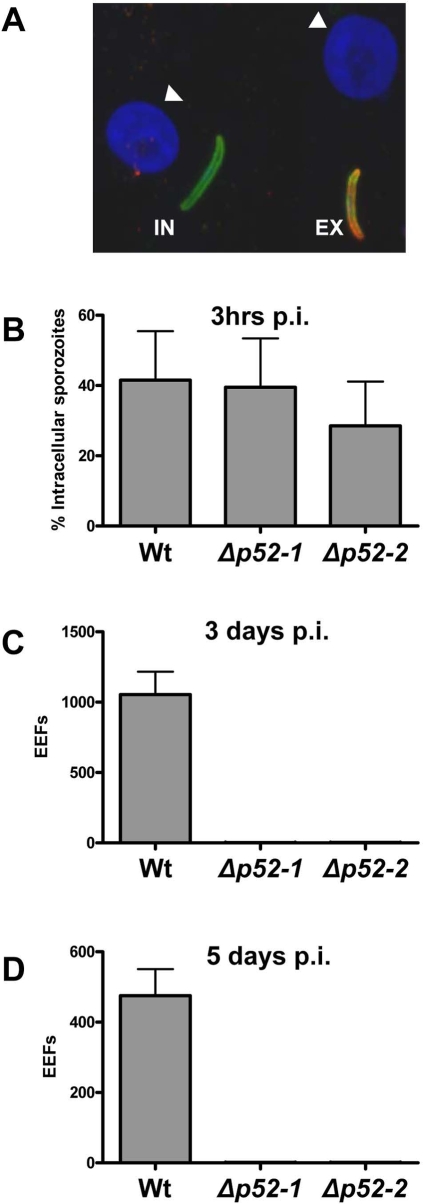
Invasion capacity of Wt and Δ*p52* sporozoites in primary human hepatocytes *in vitro*. (A) Intra (In) and extracellular (Ex) sporozoites 3 hrs after incubation of sporozoites with primary human hepatocytes in culture. Sporozoites were first stained with anti-PfCSP antibodies (red). Then cells were permeabilised and sporozoites were stained with anti-PfCSP antibodies (green). Consequently, extracellular sporozoites will stain red AND green and intracellular sporozoites will stain only green. Nuclei of the hepatocytes (white arrow heads) were stained with DAPI (B) The percentage of intracellular/invaded sporozoites (Wt and Δ*p52* mutant lines) in primary human hepatocyte 3 hours after sporozoite incubation, as determined in the double anti-CSP staining immuno-fluorescence assay (see A). (C) The number of schizonts detected by IFA using anti-HSP70 antibodies and the nuclear dye DAPI formed 3 days after incubation with either Wt or Δ*p52* mutant sporozoites. (D) The number of schizonts detected by IFA using anti-HSP70 antibodies and the nuclear dye DAPI formed 5 days after incubation with either Wt or Δ*p52* mutant sporozoites.

The ability of the Δ*p52-1* and Δ*p52-2* parasites to invade and develop inside hepatocytes was investigated using primary human hepatocytes which had been isolated directly from patient material [44]. Freshly isolated sporozoites, collected in culture medium were added to these hepatocytes that were cultured in 24 well culture plates (5×10^4^ sporozoites/well) at 37°C as previously described [44]. To examine the ability of the sporozoites to invade host cells, the infected primary human hepatocytes were fixed and examined 3 hours after incubation with sporozoites. In order to distinguish between extracellular and intracellular sporozoites, a double staining immuno-fluorescence protocol was followed [45]. Using alternatively (red and green fluorescent) conjugated anti-PFCS antibodies we stained sporozoites before and after hepatocyte permeabilisation (with 1% Triton X100). Therefore extracellular sporozoites were doubly fluorescently stained (i.e. red and green fluorescence) whereas intracellular sporozoites were only exposed to antibodies after triton X-100 treatment and were only singly fluorescently stained (i.e. green fluorescence) as can be seen in Figure 3A. In calculating the percentage of intracellular sporozoites, we found no difference in invasion of primary human hepatocytes between wild-type parasites and mutant parasites lacking *P52* (Figure 3B). To examine the intracellular parasite development to the replicating schizont stage, we analysed the parasites inside the hepatocytes after 3 days and 5 days after the addition of sporozoites. Cultures of primary human hepatocytes at either day 3 or 5 after sporozoite addition were fixed in methanol and stained using an anti-HSP70 mouse serum. Additional staining of the host and parasite DNA with DAPI, shows that wild type parasites are clearly in the process of schizogony as shown by the multiple DAPI positive nuclei. Counting of the schizonts in the culture wells revealed that at day 3 an average of 1054 liver schizonts/well are present in the cultures of the wild type parasites, however, for infections initiated with both Δ*p52* mutant lines there is a drastic reduction in the number of schizonts with an average of only 1.7 schizonts per well (Figure 3C). At day 5 the size of the wild type schizonts and the number of nuclei per schizont have increased significantly but the total number of infected cells in wild type cultures however decreased (i.e. average of 475 parasites/well) which is a well known phenomenon in *in vitro* cultures of hepatic [46]stages; where the number of infected hepatocytes decrease during the process of maturation (Figure 3D). Again, at day 5 the average number of liver schizonts formed in the infection initiated with Δ*p52* mutants is drastically reduced to 1.2 liver schizonts per well. Interestingly, the very few liver schizonts observed with the Δ*p52* mutants in day 3 and day 5 cultures have wild-type morphology with regard to both the size and number of DAPI positive nuclei. We interpret the presence of these schizonts as the result of a low contamination of wild type parasites that are the consequence of reversion events in the mutant parasite genome, resulting in the restoration of the wild-type *p52* locus (see Discussion for further details).

**Figure 4:**
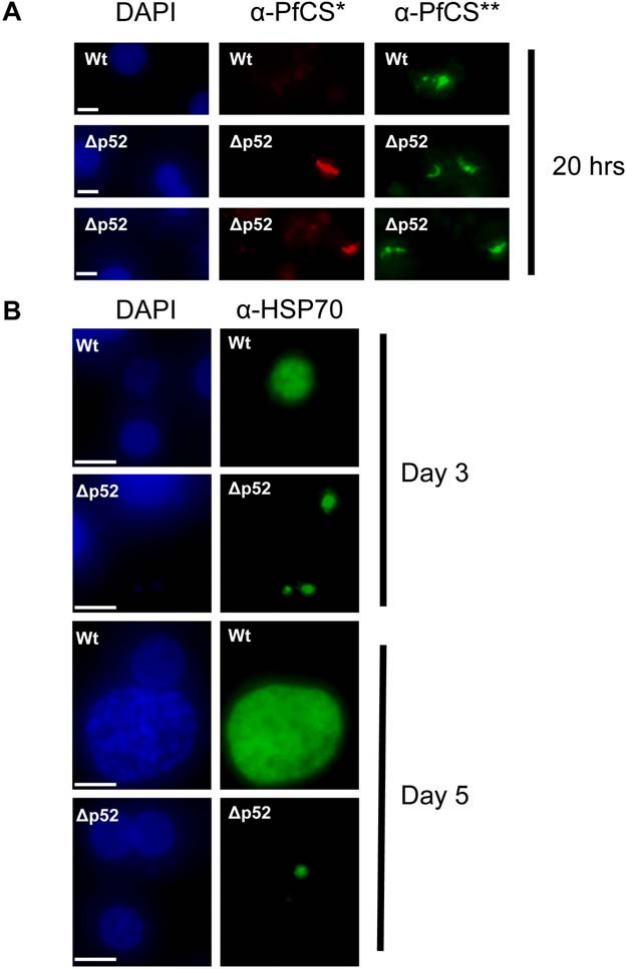
Development of wild-type and Δ*p52* parasites in primary human hepatocytes. (A) Parasites at 20 hours. Extracellular parasites are visualised by staining with anti-PfCSP antibodies (secondary conjugated with ALEXA594, i.e. red fluorescence) before permeablisation (α-PfCS*) and all parasites are visualised by staining with anti-PfCSP antibodies (secondary conjugated with ALEXA488 i.e. green fluorescence) after permeablisation (α-PfCS**). The nuclei of the host cells are stained with DAPI (blue). (B) Parasites at day 3 or day 5. Nuclei of both the host cell and the merozoites inside the developing schizont are visible by DAPI staining (blue). Parasites are identified by anti-HSP70 staining (α-HSP70; secondary antibody conjugated with ALEXA488; green). Parasite lacking P52 expression fail to develop into schizonts and the only visible forms of the parasite are small ‘rounded’, possibly degenerate and/or extracelluar, forms. Scale bars in the DAPI panels represent a size of 10 µM.

To examine the loss and aborted growth of parasites lacking P52 during development in the hepatocytes in more detail, cultures were examined at 20 hours post-infection by the double staining method used to investigate invasion (see above). At 20 hours intracellular wild-type parasites were observed to be developing inside the hepatocytes; characteristic transformation of the long slender sporozoite forms into the round trophozoites can be observed and many of these parasites are in the process of ‘rounding up’ at one end (Figure 4A). In contrast, all the visible intracellular Δ*p52* parasites appear morphologically indistinguishable from Wt parasites at 3 hours post invasion (i.e. they still maintain a sporozoite like appearance; Figure 4A). These observations show that parasites are aborted before or during the transformation of the sporozoite into the growing trophozoite stage. Further, examination of mutant parasites at either day 3 or day 5 revealed that compared to the clear liver schizont development of wild-type parasites there were very few anti-HSP70 positive parasites and those that were visible appeared to very small and round forms, which were also equally present in cultures incubated with wild-type sporozoites, possibly extracellular degraded parasites that are known to be able to persist for several days *in vitro* culture (Figure 4B). These results indicate that the Δ*p52* mutants have wild-type development up until post-hepatocyte invasion, where the mutant parasites clearly arrest soon after invasion. The intracellular parasites deficient in P52 expression maintain their slender morphology characteristics of extracellular sporozoites, whereas, wild-type parasites begin to transform into the rounded trophozoite stage by 20 hours post invasion.

## Discussion

The protein P52 belongs to the small 6-Cys family of conserved cysteine-rich proteins, many of which are membrane-anchored [21]. Several of these proteins play an important role in fertility and recognition of gametes such as P48/45, P47 and P230 [23]–[25]. These gamete surface proteins are considered to be important candidate antigens in the development of a transmission blocking vaccine. Characterization of these proteins using comparable reverse genetic technologies in rodent models of malaria and in *P. falciparum* have revealed that these proteins have similar functions in both human and rodent malaria [25], [26], [47] and van Dijk unpublished observations).

In this study we show that another member of the 6-cys family, P52, has a comparable role in both human and rodent malaria. Specifically, P52 in *P. falciparum* and its ortholog P36P in *P. berghei* function in the establishment of infection within a hepatocyte. We have previously shown that development of *P. berghei* parasites lacking P36P is aborted early after sporozoite invasion of the hepatocyte, whereas gliding motility and the capacity of these sporozoites to traverse and invade hepatocytes is not affected. We found evidence that development was aborted during or just after the formation of the parasitophorous vacuole and that the Δ*p36p* parasites had lost the capacity to prevent the host cell to undergo apoptosis [15]. Moreover, such early aborted development also occurred in the closely related rodent parasite *P. yoelii* when parasites lacked this protein [18].

In this paper we present data to demonstrate that P52 functions in *P. falciparum* at the same stage of development (i.e. intra-hepatocytic development) as its *P. berghei* ortholog. Parasites lacking P52 are not affected in their erythrocytic development (asexual or sexual) or in maturation in the mosquito. The production of sporozoites within the oocyst is not affected and the salivary glands of infected *A. stephensi* mosquitoes contain high numbers of salivary gland sporozoites (SGS) for parasites deficient in P52. This is not unexpected since large scale proteome and transcriptome analyses indicate that expression of P52 is absent in all these stages except for SGS [33], [48]. This has been further confirmed as P52 has been detected specifically in the proteome of sporozoites collected from the salivary glands and not the sporozoites from the oocyst (Lasonder et al., 2008 in press PLoS Pathogens).

The presence of proteins specific to the SGS suggests a role in sporozoite biology in the vertebrate host, anywhere along its journey to the hepatocyte, invasion of and initial intracellular remodelling of the host cell interior. For example, the SPECT1, SPECT2, TRAP and CelTOS are proteins that appear to be either exclusively present or predominantly expressed in sporozoites of the salivary gland and are present in preparation for injection into the host. These proteins have been shown to play a role in either the gliding motility of sporozoites or in cell traversal [37], [49], [50].

The sporozoites that lack P52, however, have normal gliding motility, cell traversal capacity and the ability to invade hepatocytes, which was also observed in rodent malaria parasites lacking the P36p ortholog [15], [18]. As with the rodent malaria parasite *p36p* deletion mutants, development of the *P. falciparum* parasites that lack P52, development is aborted rapidly after invasion of the hepatocyte. In the Δ*p36p P. berghei* parasites evidence was presented that the invaded parasites abort development during or just after formation of the parasitophorous vacuole. In the *P. falciparum* mutant parasites we have not observed indications of the transformation of the long slender sporozoites into the round trophozoite stage.

Perhaps not unexpectedly, we found a few parasites in the cultures of the mutant lines that were able to develop into maturing schizonts, morphologically identical to wild type schizonts. It is well known that ‘reversion-events’ can occur in the genome of mutant parasites that have been transformed with constructs that integrate by single-cross-over recombination. Such reversion events can result in removal of the integrated construct including the drug selectable marker, resulting in low levels of contamination of mutant parasite populations with wild type parasites [51]. After the feeding mosquitoes with blood containing Δ*p52* gametocytes, no drug-pressure can be applied to kill ‘revertant-parasites’ and as these mutant parasites actively multiply within the oocysts, sporozoites can be produced which restore the wild type genotype. Such ‘wild type’ parasites are the most likely explanation for the presence of the very few schizonts in hepatocytes cultured with mutant parasites. However, it remains possible that a low proportion of the mutant parasites, lacking P52 expression, are able to develop into the schizont stage. In *P. berghei* it has been shown that by infection of mice with >100000 mutant sporozoites intravenously ‘break-through’ parasites are observed that give rise to blood stage infection, despite irreversible disruption of the *p36p* gene by double cross-over recombination. Interestingly, in *P. yoelii* it has been shown that disruption of the orthologous gene *p36p* and its paralog *p36* within the same parasite, result in complete abortion of development without breakthrough parasites [18]. In *P. falciparum* the gene *p52* is in exactly the same genomic context as the rodent malaria *p36/p36p* genes and has its paralogous gene, *pf36* (PFD0210c) also immediately downstream [34]. It is therefore possible to disrupt both genes using a single DNA construct, as has been shown for other paralogous genes in rodent malaria [18], [52] and for adjacent genes encoding aspartatic proteases in *P. falciparum* [53], [54].

In infections initiated with *P. falciparum* deficient in P52 we find a greater than 99% reduction (and possibly complete absence) of EEF development very soon after sporozoite invasion. It would appear that this degree and stage of attenuation is essentially the same as described for rodent malaria parasites lacking its ortholog, *p36p*.

Consequently, P52 is the first protein in *P. falciparum* demonstrated to have an essential role at any stage of development after sporozoite invasion of the hepatocyte. Early abortion of liver stage development has also been shown for sporozoites that have been attenuated by γ-radiation (RAS). Such sporozoites are able to invade the hepatocyte but are unable to transform into the schizont stage. Invasion and establishment of an infection in the liver appears to be essential for inducing protective immune responses [10] and over-irradiated sporozoites, which are unable to initiate an infection in hepatocytes, do not induce protective immunity [55], [56]. Thus the correct dose of radiation is essential for inducing protective immune responses. We, and others, have shown that attenuated parasites generated by genetic modification (GAS) can also induce identical protective immune responses in rodent models of malaria. Genetic modification technology permits the creation of very specific and targeted alterations (deletions) in the *Plasmodium* genome as compared to the non-specific genomic or protein alterations induced by either radiation or chemical approaches. Genetic modification can therefore result in the reproducible production of homogeneous populations of parasites with a clearly defined genotype and phenotype and consequently these may have clear advantages in the testing of ‘whole parasite vaccine’ approach over RAS and CAS.

This study, showing that *P. falciparum* parasites can be attenuated by disrupting a single gene is a first, but essential, step in the development of a vaccine based on attenuated parasites. Further optimization of such parasites will likely use double cross-over recombination to avoid reversion to a ‘wild-type’ genotype; disruption of multiple genes each of which may generate arrested and/or protective parasite and thereby creating a parasite which contains successive obstacles for the restoration of parasite growth; and removal of foreign DNA from the transgenic parasite genome which can ease the transition of genetically modified organisms for human use. These are the next steps that must be accomplished before it would be possible to move such potentially protective parasites into clinical trials to test the safety, immunogenicity and potency of these parasites in immune response and re-challenge studies in humans.

## Materials and Methods

### Parasites

*P. falciparum* parasites line NF54 (wild-type; Wt) and Δ*pf52* (see below) blood stages were cultured in a semi automated culture system using standard *in vitro* culture conditions for *P. falciparum* and induction of gametocyte production in these cultures was performed as previously described [57]–[59].

### Generation of Δ*p52* parasites

The *p52* gene (PFD0215c) of *P. falciparum* was disrupted with the insertion plasmid mI44, a derivative of the previously described pDT.Tg23 plasmid [60]. The construct mI44 was generated by cloning a 1020 bp internal fragment of the *p52* coding sequence, obtained by PCR amplification using primers 1624 (5′-cgcggatccTGTAGCAATGTGATTCAAGATG) and 1625 (5′-ggactagtTGATTGTTATTATGATGTTCCTC), into the *BamH*I and *Spe*I restriction sites of the pDT.Tg23 plasmid. For details of the location of primers and sizes of amplified products see Figure 1A.

Transfection of wild type blood-stage parasites of line NF54 was performed as described [36], using a BTX electroporation system. Transfected parasites were cultured using the semi automated culture system and transformed, drug-resistant Δ*p52* parasites were selected by treatment of the cultures with pyrimethamine as described [60].

Genotype analysis of transformed parasites was performed by diagnostic PCR and Southern blot analysis. Genomic DNA of Wt or transfected parasites (blood stages or sporozoite) was isolated [61] and analyzed by PCR using primer pair 1638 (5′-CATGCCATGGTTTGAATAAGTTTTACAACCTGC) and L430 (5′-GGATAACAATTTCACACAGGA) for correct integration of mI44 in the *pf52* locus and for the presence of Wt using primer pair 1638 and 1676 (5′- GGACTAGTTTTGCCAGAATGTTCTTGTTCG), both annealing outside the target region used for integration. Primer pairs 190 (5′-CGGGATCCATGCATAAACCGGTGTGTC) and 191 (5′-CGGGATCCAAGCTTCTGTATTTCCGC) were used as a control to detect the presence of either integrated or episomal plasmid. PCR reactions were performed using the conditions as described [62]. For Southern blot analysis, genomic DNA was digested with *BstN*I and *SnaB*I, size fractionated on a 1% agarose gel and transferred to a Hybond-N membrane (Amersham) by gravitational flow [61]. The blot was pre-hybridized in Church buffer [63] followed by hybridization to a *pf52* specific probe. This probe, a PCR fragment of the coding region of *p52*, obtained with the primer pair 1624 and 1625 (see above for the sequence of these primers), was labelled using the High Prime DNA labelling kit (Roche) and purified with Micro Biospin columns (Biorad).

Cloning of transfected parasites was performed by the method of limiting dilution [64] in 96 well plates. Parasites of the positive wells were transferred to the semi-automated culture system for further genotype and phenotype analysis of the cloned parasites

### Analysis of gametocyte production

Gametocyte production was established in cultures at day 13–15 after start of the ‘gametocyte cultures’ by counting the number of mature (stage V) gametocytes in Giemsa stained thin blood films [59].

Exflagellation of male gametocytes was determined in small samples from the cultures by stimulating the gametocytes in FCS pH 8.0 at room temperature for 10 minutes. Exflagellation centres were counted under the light-microscope in 5 homogeneous fields of red blood cells at a 40× magnification.

### Analysis of mosquito stage development

14-day-old cultures of Wild-type (Wt; NF54) or Δ*p52* gametocytes were fed to *Anopheles stephensi* mosquitoes using the standard method of membrane feeding [38]. On day 7 after feeding the midguts of 40 mosquitoes were dissected and the number of oocyst counted as described [38], [65]. Statistical analysis of oocyst production (oocyst numbers) was performed with the non-parametric Wilcoxin rank-sum test.

On day 14–16 after infection, the salivary glands of the mosquitoes were collected by hand-dissection. These salivary glands were collected in William's E medium supplemented with 10% FCS, 2% penicillin-streptomycin, 1% sodium-pyruvate, 1% L-glutamine, 1% insulin-transferin-selenium (Gibco) and 10-7M dexamethasone (Sigma) and homogenized in a home made glass grinder. The free sporozoites were counted in a Bürker-Türk counting chamber using phase-contrast microscopy and the number of sporozoites per salivary gland calculated.

### Analysis of gliding motility of sporozoites

Lab-Tec 8-chamber slides (Nalge Nunc) were coated with 25 µg/ml 3SP2 antibody specific for the *P. falciparum* circumsporozoite protein (CSP) for 15 hours [40].

Sporozoites were obtained from dissection of infected *Anopheles stephensi* mosquito salivary glands. After grinding, the suspension is filtered through a 40 µm cell strainer (Falcon) to remove mosquito debris, and centrifuged at 15500 g for 3 min at 4°C. Sporozoites are then recovered in the pellet and resuspended in complete culture medium (see composition below).

Sporozoites (5×10^4^) were directly transferred to the 8-chamber slides and incubated at 37°C for 2 hours. Controls consisted in wild type sporozoites in addition a negative control consisting in WT immobilized sporozoites treated with 10 µm of cytochalasin D was also performed. Briefly, cytochalasin D (Sigma) was diluted from a 500 µM stock in Me_2_SO to a 10 µm final concentration with sporozoites. Sporozoites were then transferred to the 8-chamber slides and incubated at 37°C for 2 hours in the presence of cytochalsin D.

Sporozoites were fixed with 4% PFA and after washing with PBS, the sporozoites and the trails (‘gliding circles’) were stained with a FITC-3SP2 conjugated antibody. Slides were mounted with Vectashield and counting of the ‘gliding circles’ was performed using a DMI4000B Leica fluorescence microscope at 400× magnification. Photographs of the gliding circles were obtained with the Leica SP2 AOBS confocal microscope at the “Plate-forme d'Imagerie Cellulaire de la Pitié-Salpêtrière, Paris”.

### Cultures of primary human hepatocytes

Primary human hepatocytes were isolated from healthy parts of human liver fragments, collected during unrelated surgery in agreement with French national ethical regulations, as described . Cells were seeded in 96 well plates or 8-chamber Lab-Tec slides (Nalge Nunc) coated with rat tail collagen I (Becton Dickinson, Le Pont de Claix, France) at a density of 8×10^4^ or 21×10^4^ cells per well respectively. These cells were cultured at 37°C in 5% CO_2_ in complete William's E culture medium supplemented with 10% FCS, 2% penicillin-streptomycin, 1% sodium-pyruvate, 1% L-glutamine and 1% insulin-transferin-selenium (reagents for cell culture Gibco, Invitrogen) and 10-7M dexamethasone (Sigma, Saint Quentin Fallavier, France).

### Sporozoite cell traversal assay[66]

Hepatocyte traversal was analysed by the Dextran incorporation FACS assay [43]. HepG2-A16 (7×10^4^ cells/well) cells were seeded in 48 well plates. After 24 hours, they were incubated with 10^5^ sporozoites for 2 hours in the presence of rhodamine-dextran lysine fixable (10000MW Molecular probes, Invitrogen). After washing the cells were trypsinized, fixed with 1% formaldehyde and analyzed by FACS using a Beckman Coulter Epics xl flow cytometer. 5000 cells were counted/analysed and dextran-positive cells were detected using filter FL2 for rhodamine [43]

### Immuno-fluorescence analysis of parasite development in hepatocytes

To analyse parasite development in primary human hepatocytes, 5×10^4^ extracted sporozoites were added to primary human hepatocyte cultures, 3 hours after the addition of sporozoites, the cultures were washed with media to remove mosquito salivary gland material as well as un-invaded and un-attached sporozoites, complete media was added and cultures were incubated overnight at 37°C. The day after, the culture medium was replaced and again the 3^rd^ day post infection for cell cultures fixed at day 5 post infection [67].

Cultures with were fixed at different time points after adding the sporozoites with cold methanol and developing liver schizonts were stained with *Plasmodium* Heat shock protein 70 (HSP70) [68] followed by goat anti-mouse ALEXA-488 (Molecular probes) and nuclei were stained with 1 µg/ml diamidino-phenylindole (DAPI). For the invasion assays [45], cultures were first fixed with 4% para-formaldehyde (PFA) and extracellular (non-invaded) parasites were stained with mAbs against CSP followed by anti-mouse-ALEXA594 (i.e. red fluorescence; Molecular probes). In order to then distinguish intracellular parasites the hepatocytes were permeabilised with 1% Triton-X-100 in PBS for 4 min; allowing parasites to be stained with mAbs against CSP and these were then identified using anti-mouse-ALEXA488 (i.e. green fluorescence; Molecular probes) and nuclei were stained with 1 µg/ml DAPI. Analysis and counting of stained intracellular and extracellular parasites were performed using a DMI4000B Leica fluorescence microscope and the Olympus FluoView FV1000 confocal microscope.
